# Efficient Design of Broadband and Low-Profile Multilayer Absorbing Materials on Cobalt–Iron Magnetic Alloy Doped with Rare Earth Element

**DOI:** 10.3390/nano14131107

**Published:** 2024-06-27

**Authors:** Sixing Liu, Yilin Zhang, Hao Wang, Fan Wu, Shifei Tao, Yujing Zhang

**Affiliations:** 1 School of Electronic and Optical Engineering, Nanjing University of Science and Technology, Nanjing 210094, China; sixingliu@njust.edu.cn (S.L.); haowang@mail.njust.edu.cn (H.W.); 2 School of Materials Science and Engineering, Nanjing University of Science and Technology, Nanjing 210094, China; zhangyilin@njust.edu.cn; 3 Department of Chemistry, School of Science, Tianjin University, Tianjin 300072, China; wufan0817@tju.edu.cn

**Keywords:** cobalt–iron alloy, rare earth elements, electromagnetic absorption, multilayer absorbing materials, estimation of distribution algorithm

## Abstract

Magnetic metal absorbing materials have exhibited excellent absorptance performance. However, their applications are still limited in terms of light weight, low thickness and wide absorption bandwidth. To address this challenge, we design a broadband and low-profile multilayer absorber using cobalt–iron (CoFe) alloys doped with rare earth elements (REEs) lanthanum (La) and Neodymium (Nd). An improved estimation of distribution algorithm (IEDA) is employed in conjunction with a mathematical model of multilayer absorbing materials (MAMs) to optimize both the relative bandwidth with reflection loss (RL) below −10 dB and the thickness. Firstly, the absorption performance of CoFe alloys doped with La/Nd with different contents is analysed. Subsequently, IEDA is introduced based on a mathematical model to achieve an optimal MAM design that obtains a balance between absorption bandwidth and thickness. To validate the feasibility of our proposed method, a triple-layer MAM is designed and optimized to exhibit wide absorption bandwidth covering C, X, and Ku bands (6.16–12.82 GHz) and a total thickness of 2.39 mm. Then, the electromagnetic (EM) absorption mechanisms of the triple-layer MAMs are systematically investigated. Finally, the triple-layer sample is further fabricated and measured. The experimental result is in good agreement with the simulated result. This paper presents a rapid and efficient optimization method for designing MAMs, offering promising prospects in microwave applications, such as radar-stealth technology, EM shielding, and reduced EM pollution for electronic devices.

## 1. Introduction

With the rapid development of wireless communications and electromagnetic (EM) technologies, the intensive use of electronic devices has inevitably caused EM pollution. The pervasive propagation of EM pollution poses significant challenges, such as signal interference, data loss, and even damage to human health [1,2,3]. Therefore, EM microwave absorbing materials have attracted much attention to meet the requirement of EM radiation control in the both military and civilian fields. The demand for EM wave absorbers with a wide absorption frequency range and reduced thickness has become urgent [4,5]. EM wave absorbers can convert EM wave energy into thermal energy through magnetic or dielectric loss, which is mainly determined by the relative complex permeability or permittivity. In recent years, magnetic metal materials and their alloys have generated significant attention due to their favorable magnetic properties, including high relative complex permeability and multiple EM wave loss mechanisms of ferromagnetic resonance and eddy current effect [6,7]. Cobalt–iron (CoFe) alloys exhibit remarkable advantages, including large saturation magnetization, high Curie temperature, high permeability and easy accessibility [8,9]. However, their potential as EM-absorbing materials is limited by low natural resonance frequencies and simplistic loss mechanisms [3,10]. To overcome these limitations and enhance the absorption performance of CoFe alloys, rare earth elements (REEs), with their three unique d electrons and four localized f electrons as well as strong coupling with magnetic materials, have emerged as outstanding material dopants [11]. Recent studies have demonstrated that incorporating a certain amount of REEs such as lanthanum (La) and Neodymium (Nd) into CoFe alloys can extend the magnetic anisotropy of the CoFe alloys [12,13], reduce matching thickness and increase the resonance frequencies, which offer a promising direction for EM absorbing materials [14,15,16].

Researchers have developed several strategies to enhance the absorption bandwidth, including the utilization of multilayer structures with varying materials and thicknesses [17,18,19,20]. By implementing impedance matching, a gradient of effective permittivity and permeability can be achieved, thereby further expanding the absorption band. To address the challenging multi-objective optimization problem of determining the optimal thickness and material type of each layer in a multilayer absorbing materials (MAMs) structure, evolutionary algorithms (EAs) [21,22,23,24] such as genetic algorithm (GA), particle swarm optimization (PSO), estimation of distribution algorithm (EDA) and artificial bee colony (ABC) have been employed. In [17], a simple GA was employed to design various MAMs for predicting return loss (RL). Simultaneously, PSO was also applied to design a triple-layer microwave absorber [25]. Although the above studies show potential for optimizing absorption properties, few works were verified by experiments. Furthermore, there is still room for improvement in enhancing the performance of EAs to handle numerous variables and complex targets.

In this study, CoFe alloys are doped with La and Nd to achieve a low-profile and broadband absorber. Since the frequency range of EM waves detected by radar monitoring is generally 2–18 GHz, the EM absorption materials are designed in this frequency range. An improved EDA (IEDA) optimization is employed for the automated and efficient design of the MAMs. The absorption performance of CoFe alloys doped with different contents of La and Nd is analysed. To further broaden the absorption bandwidth, a multilayer structure is adopted to improve the impedance matching and enable multiple resonance absorption effects. Additionally, a mathematical model of the MAMs is established as an alternative to complex and time-consuming EM simulations in order to reduce simulation time while ensuring accuracy in EM performance evaluation. Consequently, IEDA is used to search for the optimal combination of thicknesses and material types from various components of La/Nd-doped CoFe alloys. To validate the effectiveness of the method, a triple-layer MAM structure exhibiting broadband absorption ranging from 6.16 to 12.82 GHz is designed. The absorption mechanisms of the triple-layer MAMs are analysed through impedance matching, energy loss and field concentration. Finally, the triple-layer MAM is further fabricated. The high agreement between experimental and numerical results demonstrates the feasibility and reliability of our proposed method. Overall, this work provides a fast and efficient design method that exhibits remarkable absorption performance in designing MAMs based on REEs-doped CoFe alloys, which offers promising potential in the field of EM absorption.

## 2. Materials and Methods

### 2.1. Preparation of CoFe Alloys Doped with Nd/La

**Figure 1:**
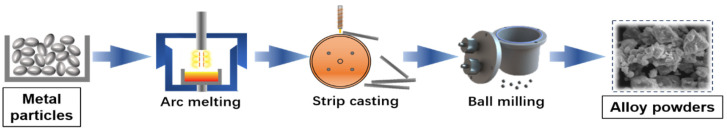
Schematic illustration of the synthesis for LCF and NCF.

The CoFe alloys, Co_0.72_Fe_0.28_, doped with Nd and La were synthesized by physical processes including arc melting, strip casting, and ball milling, as shown in Figure 1. La and Nd were used to enhance the magnetic anisotropy of CoFe alloys, which is based on our previous research [13]. Specifically, the alloys were prepared using Co (99.99 wt%, ZhongNuo Advanced Material Technology Co., Ltd., Beijing, China), Fe (99.99 wt%, ZhongNuo Advanced Material Technology Co., Ltd.), and Nd/La (99.99 wt%, ZhongNuo Advanced Material Technology Co., Ltd.) as raw materials. They were proportionally mixed and then alloyed by arc melting in the argon atmosphere. The melting state was maintained for 5 min to initiate the alloying process. To ensure a homogeneous composition of ingots, this procedure was repeated 5 times to achieve the alloying. To further characterize the ingots, they were crushed into powders in two steps. Firstly, the ingots were carefully polished to eliminate the oxide layer followed by a strip casting process where delicate alloy strips were obtained using a copper roller rotating at a fixed speed of 25 m/s. Subsequently, a mixture of 5 g prepared strips with 20 g stainless steel balls was placed in a 250 mL jar. The balling milling process lasted for 12 h at a rotation speed of 170 rpm. Finally, the Nd/La dopped CoFe alloy powders were successfully obtained. The dopant ratios of Nd and La were expressed as M_x_(Co_8_Fe_2_)_1−x_ (M = Nd, La, x = 0.075, 0.1). The collected powders were designated as follows: CF denoting the primary Co_0.72_Fe_0.28_ alloy; NCF-1/2 representing Nd_0.075_(Co_8_Fe_2_)_0.925_ and Nd_0.1_(Co_8_Fe_2_)_0.9_; LCF-1/2 indicating La_0.075_(Co_8_Fe_2_)_0.925_ and La_0.1_(Co_8_Fe_2_)_0.9_.

### 2.2. Characterization

To analyse the EM absorption properties, the samples were prepared by dispersing the powders in paraffin wax with a weight ratio of 60 wt%. The EM parameters (relative permeability and relative permittivity) between 2 and 18 GHz were measured by a vector network analyser (VNA, Agilent N5244A, Santa Clara, CA, USA).

## 3. Design and Optimization of Multilayer Absorber Materials

### 3.1. Mathematical Model of the Multilayer Absorbing Materials

**Figure 2:**
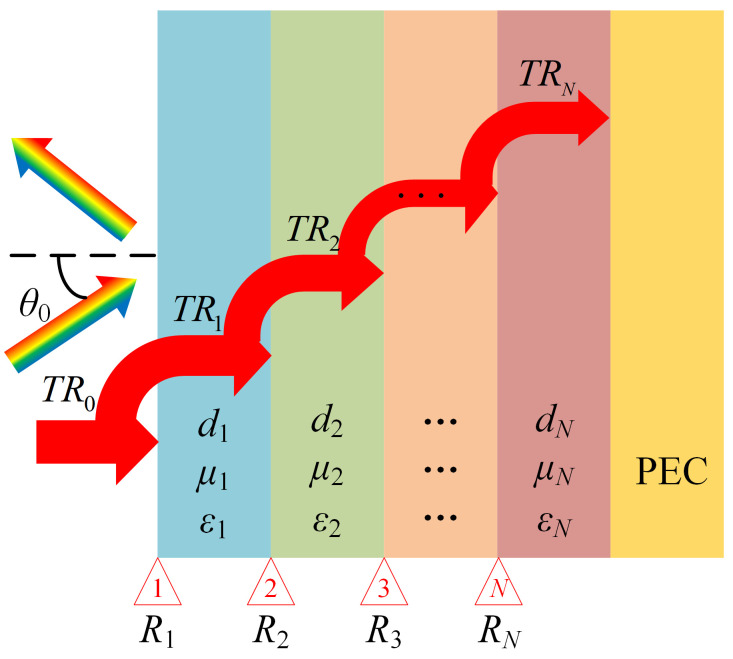
Schematic of the model of a MAM design with *N* layers of different materials. *d_i_*, *μ_i_* and *ε_i_* denote the thickness, relative permeability, and relative permittivity of the ith layer, where *i* represents the layers recursively as *i* = 1, 2, …, *N*. *i* = 0 indicates the air. It is supposed that the incident wave propagates from the air at an incident angle of *θ*_0_.

The propagation of EM waves in a MAM design involves reflection and transmission across different layers with varying mediums. Figure 2 illustrates the mathematical model of a multilayer absorber consisting of *N* layers with different materials, backed by a perfect electric conductor (PEC). The total RL of a MAM design (*TR*_0_) can be obtained by a recursive formulation [26]. The computation of the total RL considers the multiple reflections (*R*_1_, *R*_2_, …, *R_N_*) at each interface, which can be recursively calculated by:

(1) $$TR_{i}=\frac{R_{i}+TR_{i+1}e^{-2jk_{i,z}d_{i}}}{1+R_{i}TR_{i+1}e^{-2jk_{i,z}d_{i}}},$$

where the wave number is $k_{i,z}=\cos\theta_{i}\omega \sqrt{\mu_{i}\varepsilon_{i}}$ and ω=2πf is the angular frequency. f is defined as the operation frequency. According to Snell’s law [27], the incident angle of the EM wave propagates into each interface is as follows:

(2) $$\frac{\sin\theta_{i}}{\sin\theta_{i-1}}=\sqrt{\frac{\mu_{i-1}\varepsilon_{i-1}}{\mu_{i}\varepsilon_{i}}}.$$

In this model, the RL at each interface is expressed as:

(3) $$R_{i}=\frac{\mu_{i}k_{i-1,z}-\mu_{i-1}k_{i,z}}{\mu_{i}k_{i-1,z}+\mu_{i-1}k_{i,z}}.$$

As *θ*_0_, *μ_i_*, *θ_i_* and *ε_i_* are generally known and the reflection coefficient of the PEC layer is *TR_N_* = −1, the total RL of the MAMs *TR*_0_ at 2–18 GHz can be derived.

### 3.2. Optimization Using IEDA for Searching the Broadband and Low-Profile Structure

**Figure 3:**
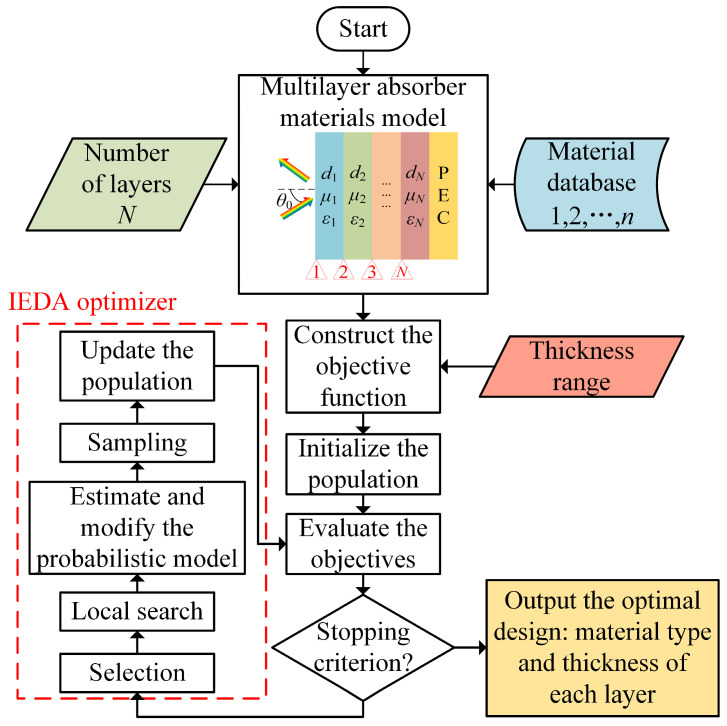
The framework of optimization process for a MAM design.

The optimization process of the MAM design based on IEDA is shown in Figure 3. The process is mainly composed of three parts: EM modelling of the MAM design, construction of the objective function, and optimization using IEDA. In terms of the EM modelling for multilayer absorber materials design, some physical parameters need to be predefined, including the number of layers *N* and the source of the material database. Subsequently, the objective function should be determined depending on the performance requirements.

EDA [28] has been developed to guide the search for the global optimum by estimating and sampling the probabilistic model of promising solutions. Over the past few decades, EDA has been extensively applied to solve complex problems [29]. In order to improve the efficiency of EDA, the improved estimation of distribution algorithm (IEDA) is introduced in this article. Firstly, a randomly generated initial population is evaluated by the objective function. If the stopping criterion is met, the optimization process terminates and outputs the optimal solution with specified material type and thickness for each layer. Otherwise, individuals with better objective function values are selected from the population. Next, a competitive neighbourhood search [30] is adopted to locally explore promising individuals in two neighbourhoods surrounding these selected individuals, fully leveraging information from the probabilistic model. Subsequently, based on these promising individuals, we estimate the probabilistic model [28]. To effectively improve population diversity, a two-level correlation [23] is used to modify the probabilistic model. Sampling individuals are then randomly drawn according to the modified probabilistic model. Finally, both promising individuals and sampling individuals constitute the new population. In this way, IEDA achieves a balance between local exploitation and global exploration.

## 4. Results and Discussion

### 4.1. Effect of Doping La/Nd on the Absorption Performance of CoFe Alloys

**Figure 4:**
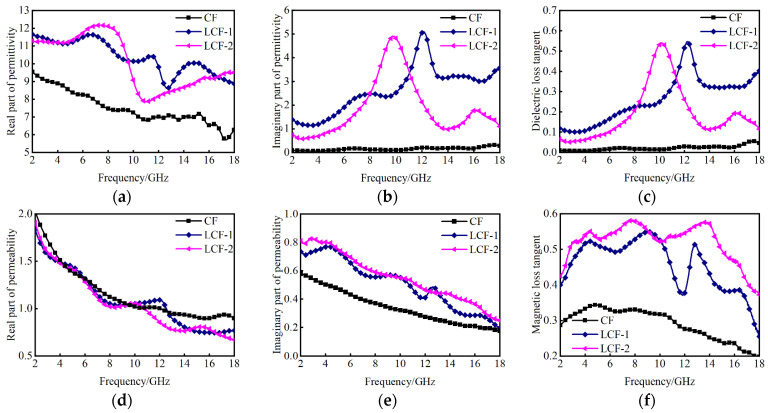
The measured EM parameters of CF, LCF-1 and LCF-2 in the concerned bands: (**a**) the real part of the permittivity; (**b**) the imaginary part of the permittivity; (**c**) dielectric loss tangent; (**d**) the real part of the permeability; (**e**) the imaginary part of the permeability and (**f**) magnetic loss tangent.

Typically, the relative permittivity (ε) and relative permeability (μ) can be utilized to evaluate the EM absorption performance of materials [31]. The real and the imaginary parts of ε and μ represent the energy storage and dissipation capabilities of EM waves, respectively. The loss tangent value can illustrate the attenuation capability. Figure 4 illustrates the measured EM parameters of CF, LCF-1 and LCF-2 within a frequency range of 2 to 18 GHz. In Figure 4a, the curve depicting the real part of permittivity (ε′) exhibits a decreasing trend with increasing frequency for LCF-1 and LCF-2. The ε′ values of CF, LCF-1 and LCF-2 vary in the range of 5.7–9.6, 8.6–11.7 and 7.8–12.2, respectively. The introduction of La doping induces significant modifications in the ε′ values of CF accompanied by a pronounced resonance peak in polarization leading to an augmentation in dielectric loss. From Figure 4b, the imaginary part of the permittivity (ε″) values for CF are close to 0. The ε″ values fluctuate in the range of 1.1–5.2 for LCF-1 and 1.1–7.3 for LCF-2. LCF-2 exhibits the biggest ε″ values, which indicates that LCF-2 has a higher dielectric dissipating property. Both LCF-1 and LCF-2 exhibit a resonance peak for ε″, implying the intense polarization relaxation in the powders. The dielectric loss tangent (tanδe = ε″/ε′) values exhibit a similar trend to the ε′ values shown in Figure 4c. The curve of CF almost has no change. With the doping of La, the peak tanδe values were observed for LCF-1 and LCF-2 at 12.08 GHz and 9.84 GHz, indicating good dielectric loss. As depicted in Figure 4d and e, μ′ values of CF, LCF-1 and LCF-2 all vary in the range of 0.6–2.0, which illustrates that the doping of La has little effect on the real part values of permeability (μ′) for CoFe alloys. The imaginary part values of permeability (μ″) values fluctuate in the range of 0.1–0.8 for both LCF-1 and LCF-2. With the increase in La doping, μ′ exhibits a decreasing trend, while μ″ demonstrate an increasing trend, thereby contributing to higher magnetic loss. The plot in Figure 4f illustrates the variation in the magnetic loss tangent (tanδm = μ″/μ′) values. It is evident that the magnetic loss values increase with the increase in the La component, which is mainly because the increased doping of the La phase might bring strengthened magneto crystalline anisotropies, hence hoisting the natural resonance frequencies and magnetic loss.

**Figure 5:**
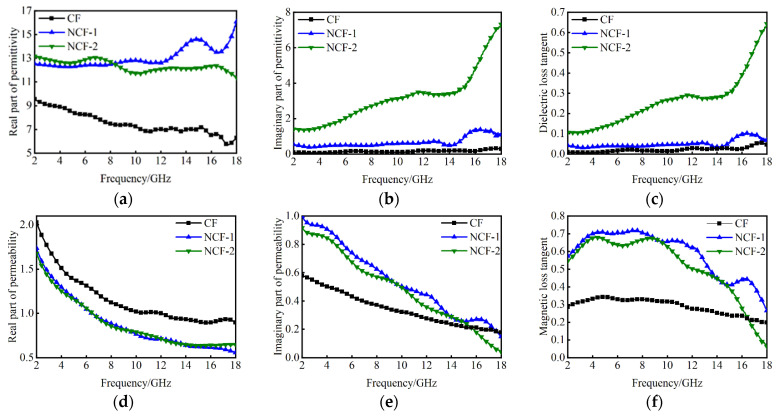
The measured EM parameters of CF, NCF-1 and NCF-2 in the concerned bands: (**a**) the real part of the permittivity; (**b**) the imaginary part of the permittivity; (**c**) dielectric loss tangent; (**d**) the real part of the permeability; (**e**) the imaginary part of the permeability and (**f**) magnetic loss tangent.

Figure 5 shows the values of ε and μ for CoFe alloys doped with different Nd contents in the frequency range from 2 GHz to 18 GHz. In Figure 5a, ε′ values of NCF-1 and NCF-2 vary in the range of 12.2–16.1 and 11.3–13.2, respectively. In Figure 5b, ε″ values vary in the range of 0.3–1.4 and 1.3–7.4 for NCF-1 and NCF-2, respectively. However, despite NCF-2 having more Nd content, ε′ and ε″ values of NCF-2 are lower than those of NCF-1. It indicates that an appropriate Nd doping strategy can effectively enhance the conductivity of CF. In addition, the tan*δ*_e_ values follow a similar trend as the ε′ values in Figure 5c. Although *μ*′ values of NCF-1 and NCF-2 decrease, *μ*″ values have an increasing trend with the doping of Nd content from Figure 5d,e, which implies the improvement of magnetic loss, as the curves shown in Figure 5f.

**Figure 6:**
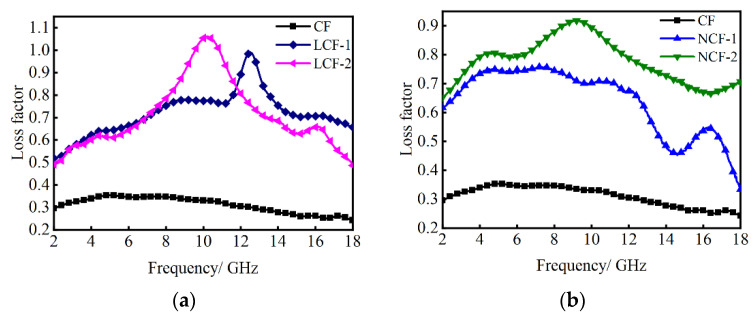
Loss factor of (**a**) CF, LCF-1 and LCF-2 and (**b**) CF, NCF-1 and NCF-2.

Figure 6 shows the loss factor tan*δ* (tan*δ* = tan*δ*_e_ + tan*δ*_m_) of CF, LCF-1/2 and NCF-1/2 in the frequency of 2–18 GHz. The curve representing CF exhibits the lowest values compared to LCF-1/2 and NCF-1/2, which indicates poor EM loss capability. From Figure 6a, it is evident that an increase in La content leads to a significant improvement in the tan*δ* values of LCF, suggesting a notable enhancement in their EM loss ability. Specifically, LCF-1 and LCF-2 exhibit maximum tan*δ* values of 0.99 at 12.52 GHz and 1.06 at 10.24 GHz, respectively, indicating that LCF-1/2 exhibits a superior loss effect in the high-frequency region. As seen in Figure 6b, when the Nd content is doped, the tan*δ* value of NCF has a great improvement. Notably, NCF-2 demonstrates a superior loss effect compared to NCF-1 with a maximum loss factor of NCF-2 reaching up to 0.92 at 9.04 GHz. It can be found that the loss tan*δ* of CoFe alloys is enhanced when La and Nd are doped, which could be caused by the high mobility of the three Nd/La itinerant d electrons and four localized f electrons [32]. As a result, the enhanced dielectric and magnetic losses of LCF and NCF render them highly promising materials for absorption applications.

**Figure 7:**
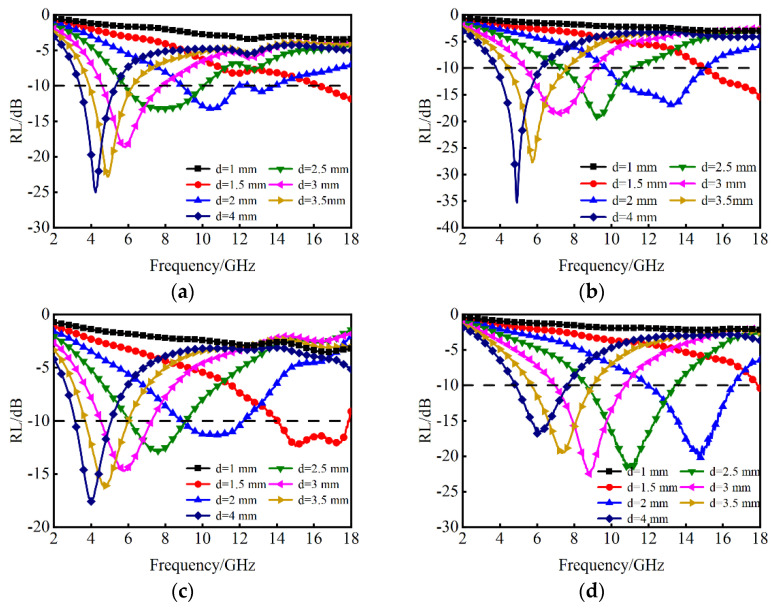
Frequency dependence of RL for (**a**) LCF-1; (**b**) LCF-2; (**c**) NCF-1 and (**d**) NCF-2 with different thicknesses.

The relationship curves of RL and frequency of Nd/La-doped CoFe samples with varying thicknesses are presented in Figure 7. The absorption performance of the samples significantly is improved with increasing doping of La and Nd content. In comparison to NCF samples, LCF samples exhibit superior absorption performance in both low-frequency and mid-to-high-frequency bands. All LCF samples demonstrate better RL values in the low-frequency band for all thicknesses, while also exhibiting a wider absorption bandwidth in the mid-high frequency bands. The dopant of La and Nd can increase the magnetic anisotropy of the CoFe alloys, thereby increasing the magnetic natural resonance frequency. As a result, the absorption frequency band of La/Nd-doped CoFe alloys can be extended to higher frequencies. As the thickness values increase, the minimum RL values and their corresponding frequencies decrease accordingly. However, it can be seen that the absorption bandwidths of samples are not improved with the increasing of the thicknesses, indicating that there is no linear relationship between the absorption bandwidth and the thickness. Due to the single-layer structure, the LCF and NCF samples would operate at around the central frequency of the wavelength and suffer from narrow bandwidth. To broaden the absorption bandwidth and ensure a low-profile structure, a MAM structure is designed and optimized. Different from the single-layer MAMs, the multilayer structure of absorbing materials can not only achieve 1/4 wavelength (*λ*/4) resonance absorption but also increases the interface between materials and free space, which has been proved to be an efficient strategy for achieving broadband absorption [33,34]. In this study, we take LCF and NCF with favorable EM parameters as the material database and improve the impedance matching by optimizing the thickness and material type of each layer of the MAMs. This approach integrates multiple loss mechanisms to achieve flexible regulation of EM waves.

### 4.2. MAMs Design Using IEDA Optimizer

**Figure 8:**
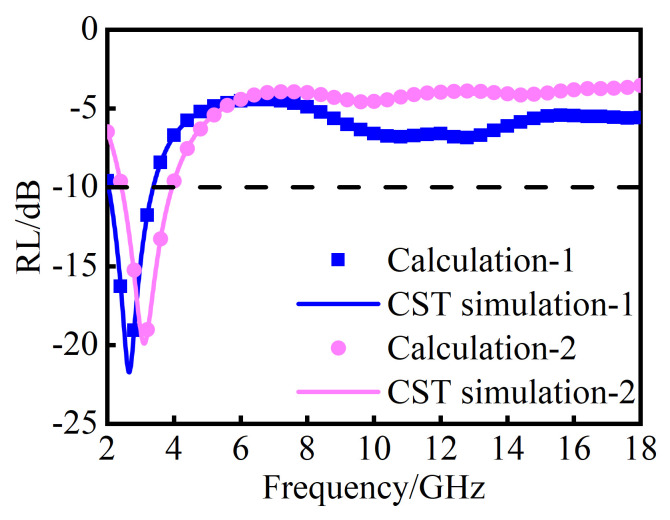
Comparison of simulation and calculation results using CST and mathematical model, respectively.

Generally, the absorption performance of a MAM design is simulated by EM simulation software, such as CST Microwave Studio 2021 (release version is 2021. 01), which provides accurate results but requires significant time investment. In CST Microwave Studio, field monitors and post-processing templates are incorporated to obtain the total RL of MAMs through the Finite Integration method [35]. In contrast, the mathematical model of multilayer absorber materials design can effectively calculate the RL by Equation (1), thereby enhancing optimization efficiency. To validate the feasibility of the mathematical model in Section 3.1, the calculated results by Equation (1) are compared with the simulated results conducted in CST software. From Figure 8, the calculated results are highly coincident with the simulated results. It can be seen that the mathematical model can replace the EM simulation to achieve the rapid evaluation of the absorption performance of the MAMs.

In order to validate the availability of the proposed optimization method, a triple-layer MAM (*N* = 3) is designed, with materials selections from NCF-1/2 and LCF-1/2. Table 1 shows the relevant parameters. The thickness range of each layer is defined ranging from 0.45 mm to 3 mm. The material type (*m_i_*) and thickness (*d_i_*) for each layer are encoded as binary code, which are written as:

(4) $$\left\{\begin{array}{l} m_{i}=m_{i2}\times 2+m_{i1}\times 1+1\\ d_{i}=\left(\begin{array}{l} d_{i8}\times 128+d_{i7}\times 64+d_{i6}\times 32+d_{i5}\times 16\\ +d_{i4}\times 8+d_{i3}\times 4+d_{i2}\times 2+d_{i1}\times 1 \end{array}\right)\times 0.01+0.45 \end{array}\right.,$$

where *m_i_*_1_~*m_i_*_2_ and *d_i_*_1_~*d_i_*_8_ are binary codes. The material database consists of four materials, with thicknesses categorized based on the range. Consequently, a 30-bit binary encoding is employed to represent the decision variables. The objective of this article is to obtain a triple-layer MAM that exhibits the widest relative frequency band where the RL is below −10 dB and possesses the lowest relative thickness. It is a multi-objective optimization and can be described as:

(5) $$\min:fitness=-\mathrm{FBW}/d_{r},$$

where FBW and *d*_r_ are the relative bandwidth with the RL below −10 dB and the relative thickness. Meanwhile, FBW and *d*_r_ are given as

(6) $$\mathrm{FBW}=2\times \left(f_{H}-f_{L}\right)/\left(f_{H}+f_{L}\right),$$

(7) $$\mathrm{and}\;d_{r}=\left(d_{1}+d_{2}+\ldots +d_{N}\right)/\left(\lambda_{L}-\lambda_{H}\right),d_{i}\in \left[0.2,3\right],$$

where $f_{L}$ and $f_{H}$ are the low and high limits of the frequency range with the RL below −10 dB. The wavelength of $f_{L}$ and $f_{H}$ are $\lambda_{L}=c/f_{L}$ and $\lambda_{H}=c/f_{H}$, where *c* is the velocity of light in free space.

**Figure 9:**
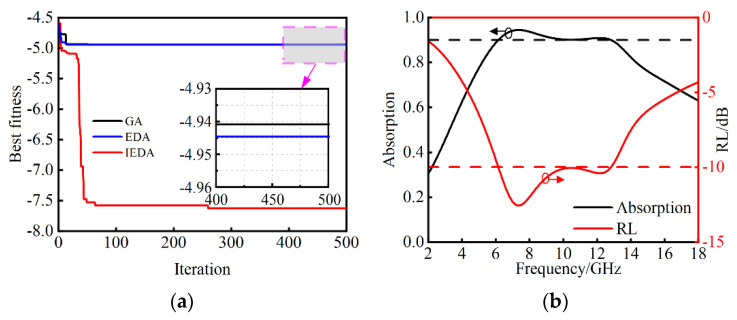
(**a**) Converge curves of the best fitness over the number of iterations for GA, EDA and IEDA. (**b**) The RL and absorption results obtained by IEDA.

IEDA is compared with GA and EDA to demonstrate the superiority of the proposed method, and the evolution of the iterations can be seen in Figure 9a. The optimal fitness achieved by IEDA (−7.80) outperformed that obtained by EDA (−4.94) and GA (−4.90), indicating that IEDA converges faster and explores better solutions. These results validate the feasibility and efficiency of our proposed optimization method. The RL and absorption results of the optimal triple-layer MAMs obtained by IEDA are described in Figure 9b. The optimized triple-layer MAMs cover the C and X bands ranging from 6.16 GHz to 12.82 GHz. Table 2 shows the optimization parameters of the optimal structure, the material types of each layer are LCF-2/, LCF-1/LCF-2 from the first and third layers, respectively. By employing the proposed optimization method, a remarkable relative bandwidth of 70.18% and a relative thickness of 0.09 are achieved.

**Figure 10:**
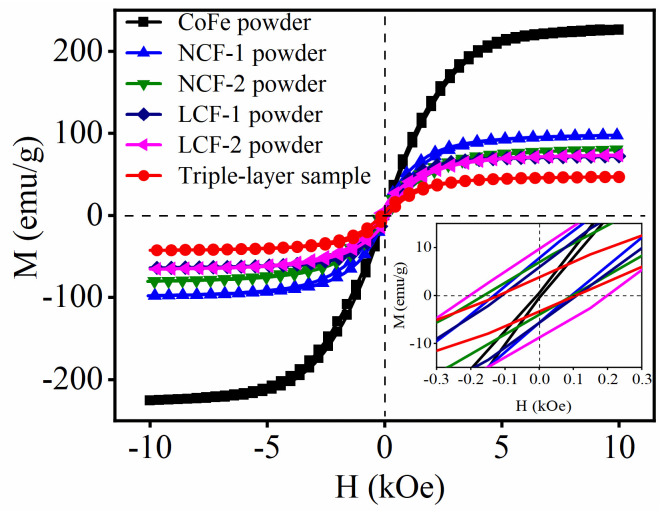
Magnetic hysteresis loops for CoFe alloys, LCF-1/2, NCF-1/2 and the triple-layer sample.

The magnetism plays a crucial role in determining the microwave loss, as it is essential for the typical magnetic phases. Figure 10 presents the measured saturation magnetization (M_s_) and coercivity (H_c_) at room temperature. As observed, the CoFe alloy has high M_s_ but low H_c_ values. The dopant of Nd/La leads to noticeable reductions in M_s_ but significant increases in H_c_ for the NCF/LCF samples. M_s_ reflects the alignment of magnetic domains within the magnets, while H_c_ represents their resistance to alternate EM fields, resulting in higher energy consumption required for realignments. This aligns with variations observed in real/imaginary permeability trends, where Nd- and La-doped samples exhibit higher μ″ values and enhanced magnetic loss characteristics in Figure 4 and Figure 5. Furthermore, the triple-layer MAMs also maintain the magnetic properties of LCF samples. The slight decrease in M_s_ could be attributed to binder-induced effects from paraffin without compromising H_c_ values. Therefore, even with triple-layer MAM samples, potent magnetic loss is preserved.

**Figure 11:**
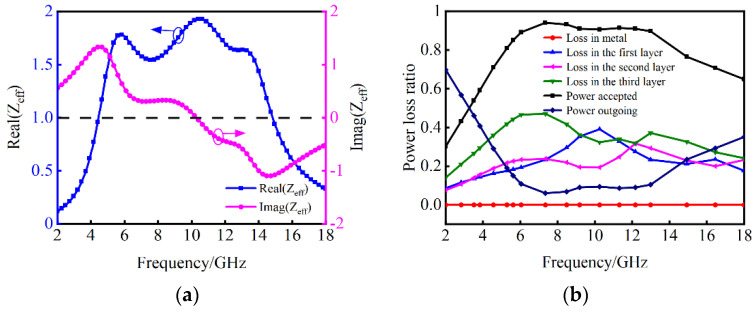
(**a**) Impedance matching of the triple-layer MAMs. (**b**) Power loss ratio of each layer in the triple-layer MAMs.

**Figure 12:**
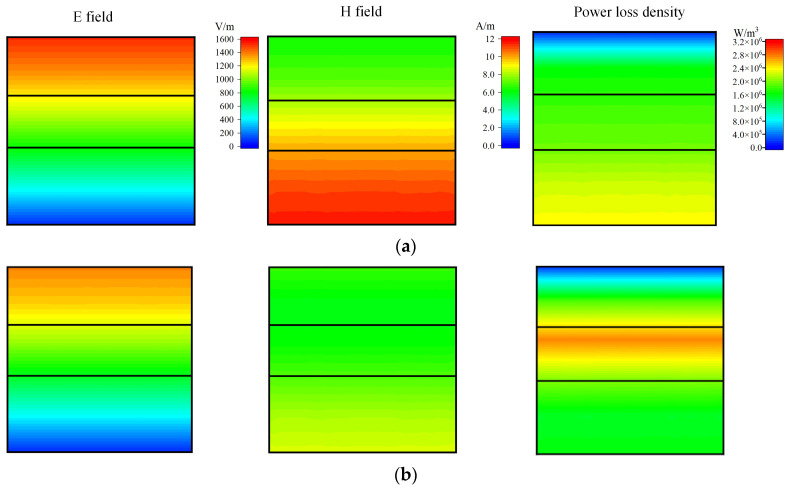
Distributions of electric field (E field), magnetic field (H field) and power loss density of the triple-layer MAMs (**a**) at frequency f=7.344 GHz and (**b**) at frequency f=12.176 GHz.

To further explain the necessity of the impedance matching design, the equivalent impedance of the triple-layer MAMs is calculated by:

(8) $$Z_{\mathrm{eff}}=\sqrt{\frac{{\left(1+S_{11}\right)}^{2}-{S_{21}}^{2}}{{\left(1-S_{11}\right)}^{2}-{S_{21}}^{2}}},$$

where *S*_11_ and *S*_21_ are the reflectance and transmittance of the absorber. Figure 11a shows the impedance matching between the triple-layer MAMs and free space. From 6.16 GHz to 12.82 GHz, the real part of the equivalent impedance (Real(*Z*_eff_)) is close to one and the imaginary part of the equivalent impedance (Imag(*Z*_eff_)) fluctuates around zero, implying excellent impedance matching with free space and facilitating efficient entrance of incident EM waves into the triple-layer MAMs. To illustrate the EM energy loss mechanism of the triple-layer absorber, the power loss ratio of each layer in the triple-layer MAMs is analysed in Figure 11b. It can be seen that the PEC does not absorb energy. The second layer of LCF-1 exhibits the smallest absorption power ratio, ranging from 7.58% to 31.67%. From 2 GHz to 18 GHz, significant energy absorption occurs in the third layer of LCF-2. The average absorption power ratio of the third layer is 33.52%, with a maximum power loss ratio of 47%. The first layer of LCF-2, adjacent to the free space, has an average absorption power ratio of 21.78% and a maximum power loss ratio of 39.05%. Although the first layer and the third layer are both composed of LCF-2 material, their absorption properties differ significantly due to the variations in thicknesses that should be carefully designed. The first and second layers exhibit similar power loss ratios at 2–7 GHz as well as 12–18 GHz, differing only by a range of 0.4–4.3%. From 11 GHz to 18 GHz, the second and third layers demonstrate comparable power loss ratios. In other frequency bands, the third layer exhibits a higher absorption power ratio compared to the other two layers. Through the synergistic absorption effects among material layers, the designed triple-layer MAMs achieve wideband absorption performance by absorbing over 90% of EM wave energy within the frequency range of 6.16–12.82 GHz. It is evident that there are two absorption peaks at 7.344 GHz and 12.176 GHz which correspond to the RL and absorption results in Figure 9a. To further investigate the absorption mechanism of the triple-layer MAMs, the simulated distributions of the electric field (E filed) and magnetic field (H field) and power loss density are shown in Figure 12. At a low frequency, such as 7.344 GHz from Figure 12a, the distribution curves of E and H fields form a separation in the third layer next to the PEC, which exhibits the occurrence of standing wave and exerts the typical quarter-wave (*λ*/4) resonance. The power loss density and the H field both concentrate on the third layer, which manifests *λ*/4 magnetic resonance. It shows that most of the energy is consumed by the third layer, as discussed in Figure 11b. As the frequency increases to 12.176 GHz, the skin depth decreases. From Figure 12b, the E field is primarily localized within the first and second layers, as well as at their interface, which demonstrates multiple reflections of EM waves occur in the adjacent layers and thus promotes the dissipation of the EM energy. The distribution of the H field is mainly concentrated at the third layer and the interface of each layer. Compared with the distribution of E and H fields in the third layer at 7.344 GHz, there is an increase in both E field intensity and H field intensity in the second layer as well as at each interface. The power loss distribution reveals a notable enhancement at the interface between the first and second layers, indicating a synergistic effect arising from both dielectric and magnetic losses. In conclusion, the various loss mechanisms in the La-doped CoFe alloy (LCF materials) contribute to EM energy dissipation, while the multilayer structure enhances impedance matching and controls field distribution. Consequently, broadband absorption of the triple-layer MAMs is achieved through the combined effects of impedance matching, energy loss and field concentration.

### 4.3. Experimental Verification

**Figure 13:**
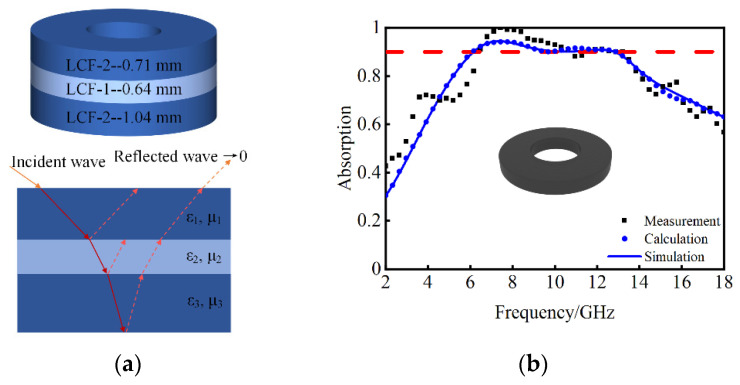
(**a**) The schematic diagram of the triple-layer sample. (**b**) Comparison of measurement, calculation and simulation results for the triple-layer MAMs.

After determining the geometric parameters and material type, a triple-layer sample was prepared for verification in this study. Each layer of the sample was separately obtained and pressed into the coaxial waveguide cavity to obtain the triple-layer MAMs, as shown in Figure 13a. The sample ring had an outer diameter of 7.0 mm, an inner diagram of 3.0 mm and a thickness of 2.39 mm for microwave reflection measurement. The coaxial method was used to obtain the reflection of the sample in the VNA. Figure 13b shows the simulated, calculated and measured results, which exhibit excellent agreement between the simulation and measurement curves with minor discrepancies attributed to the limitation in measurement techniques and manufacturing errors during fabrication processes. The calculation and simulation curves are in good consistency, which further demonstrates the feasibility of our proposed method. In summary, our proposed optimization method enables a broadband absorption performance with low-profile MAMs. Table 3 presents a comparison between our triple-layer MAMs with other relevant works in recent years for EM wave absorption. Notably, in comparison to the single-layer absorber utilizing CoFe materials [36], the multilayer absorber [20], two absorbers incorporating REEs [7,14], and the proposed low-profile and broadband MAMs have broader bandwidth and lower relative thickness, indicating the feasibility of this design method in the EM absorption field.

## 5. Conclusions

In this work, an efficient design method for broadband and low-profile MAMs based on La/Nd-doped CoFe alloys is proposed. The effects of the La/Nd content on the absorption performance of CoFe alloys are analysed, revealing that the excellent dielectric and magnetic loss of LCF and NCF make them promising candidates for EM absorption materials. To further enhance the absorption bandwidth, a mathematical model of the MAMs is established to replace complex and time-consuming EM modeling. The agreement between calculation and simulation results demonstrates the reliability of this mathematical model. Additionally, an IEDA is introduced to simultaneously optimize thickness and RL below −10 dB of the MAMs. To verify the proposed method, a triple-layer MAM is designed with a wide absorption bandwidth covering C, X, and Ku bands (6.16–12.82 GHz) and a total thickness of 2.39 mm. The absorption mechanism of the triple-layer MAMs is systematically investigated, showing that various loss mechanisms in La-doped CoFe alloy (LCF materials) contribute to EM energy dissipation while the multilayer structure enhances impedance matching and controls field distribution. Finally, a triple-layer ring prototype based on the designed triple-layer MAMs is fabricated. The good agreement between simulation and experimental results confirms the efficiency of our proposed method. The designed MAMs hold great potential in radar-stealth technology development, EM shielding for health safety as well as reduced EM pollution for electronic devices. The proposed design method can be extended to other absorbing materials, such as ferromagnetic materials [37,38], offering promising prospects for EM wave absorption.

## Figures and Tables

**Table 1:** Material database for the triple-layer MAMs.

Material Name	Supplementary Notes	Material Name	Label
La*_x_*(Co_8_Fe_2_)_1−*x*_	x = 0.075	LCF-1	1
	x = 0.1	LCF-2	2
Nd*_x_*(Co_8_Fe_2_)_1−*x*_	x = 0.075	NCF-1	3
	x = 0.1	NCF-2	4

**Table 2:** Optimization parameters of the triple-layer MAM design.

Structure	Material	Thickness (mm)	Total Thickness (mm)
Triple-layer MAMs	*m*_1_ = LCF-2	*d*_1_ = 0.71	2.39
	*m*_2_ = LCF-1	*d*_2_ = 0.64	
	*m*_3_ = LCF-2	*d*_3_ = 1.04	

**Table 3:** Comparison with other related work.

Reference	Materials	Structure	FBW	EAB (GHz)	Operating Frequency	Relative Thickness	Total Thickness (mm)	Fitness
[36]	CoFe/Go composite	Single-layer	36.19%	8.6–12.4	X, Ku	0.47	5	−0.77
[20]	CoFe-C alloy	Triple-layer	59.56%	7.9–14.6	C, X, Ku	0.20	3.5	−2.98
[14]	CoZn ferrites doped with Pr	Single-layer	50.96%	10.69–18	X, Ku	0.22	2.5	−2.32
[7]	Bi doped LaFeO_3_	Single-layer	30.01%	10.28–13.91	X, Ku	0.29	2.18	−1.03
This work	CoFe alloy doped with La	Triple-layer	70.18%	6.16–12.82	C, X, Ku	0.09	2.39	−7.80

## Data Availability

Data are contained within the article.
